# Preparation and Characterization of Graphene Oxide/Polyaniline/Carbonyl Iron Nanocomposites

**DOI:** 10.3390/ma15020484

**Published:** 2022-01-09

**Authors:** Yun-Yun Huang, Jian Wu

**Affiliations:** College of Chemical Engineering, Fuzhou University, Fuzhou 350108, China; N190420034@fzu.edu.cn

**Keywords:** carbonyl iron powder, polyaniline, graphene oxide, wave absorbing, anti corrosion

## Abstract

Nano coatings for anti-corrosion and electromagnetic wave absorbing can simultaneously implement the functions of assimilating electromagnetic waves and reducing the corrosion of materials caused by corrosive environments, such as seawater. In this work, a composite material for both electromagnetic wave absorption and anti-corrosion was prepared by an in-situ chemical oxidation and surface coating method using carbonyl iron powder (CIP), graphene oxide (GO) and aniline (AN). The synthesized composite material was characterized by scanning electron microscopy (SEM), infrared spectroscopy (FT-IR) and XRD. The carbonyl iron powder-graphene oxide-polyaniline (CIP-GO-PANI) composite material was used as the functional filler, and the epoxy resin was the matrix body for preparing the anticorrosive wave-absorbing coating. The results show that CIP had strong wave-absorbing properties, and the anti-corrosion property was greatly enhanced after being coated by GO-PANI.

## 1. Introduction

Electromagnetic waves can be interfered with by objects through conduction coupling and electromagnetic field coupling. It is difficult to protect against this, and it is also harmful to metal materials. The wave-absorbing material has gradually become a research hotspot. Corrosion can cause great harm to materials and waste resources. The annual loss due to corrosion in many countries accounts for 3% to 5% of GDP, and the corrosion loss in China has reached 3.44% of its GDP. The steel wasted every year due to corrosion accounts for 30% of the total production, half of which can be avoided by applying modern anti-corrosion technology. Therefore, seeking non-toxic, bio-environmental coatings with anti-corrosion and wave-absorbing properties is of great significance to the sustainable development of society.

In recent years, the application of GO/PANI/CIP nanocomposites in anti-corrosion and electromagnetic wave absorption has been reported. Mohammadi et al. [1] used graphene as a filler to be added into epoxy resin composite coatings. They studied the influence of graphene filler content on the barrier effect and found that the anti-corrosion performance was the best at 0.5% content. However, excessive graphene is prone to agglomeration, which is not conducive to the barrier effect of the coating on the corrosive medium. 

Di et al. [2] used Fe_3_O_4_ to modify GO. The Fe_3_O_4_ nanoparticles were deposited on the surface of graphene oxide. Compared with the unmodified sample, Fe_3_O_4_-GO was more uniformly dispersed in the epoxy resin, which significantly improved the overall performance of the composite coating. Pan et al. [3] prepared water-based polyaniline and blended it with epoxy resin to prepare a composite coating. They found that the anti-corrosion performance of the composite coating was significantly higher than that of the bare steel coated with pure epoxy. 

You et al. [4] used iron nanosheets as a wave absorber and found that when the coating thickness was 3.0 mm, the maximum RL reached −45.0 dB, and the absorption bandwidth below −10.0 dB was 4.0 GHz. Fan et al. [5] prepared CIP/PANI core-shell wave-absorbing composite materials by in-situ polymerization. When the CIP/PANI ratio was 0.2:1 and the coating thickness was 1.4 mm, the bandwidth of RL < −10 dB was 10.9–16.7 GHz, and the maximum RL was −52.1 dB. 

Tang et al. [6] prepared PANI-coated CIP composite materials and found that they had good corrosion resistance and microwave absorbing properties. The RL at 27.3–39.5 GHz and the coating thickness of 1.1 mm were all better than −10 dB. Xu et al. [7] loaded RGO on the surface of CIP, and coated PANI on the surface through in-situ polymerization to obtain a core-shell structure of RGO/CIP/PANI composite material. The minimum reflectance of the prepared coating reached −38.8 dB at the thickness of 2 mm. 

In this work, a composite with both absorbing electromagnetic wave and anti-corrosion function was prepared by an in-situ chemical oxidation and surface coating based on previous studies using carbonyl iron powder (CIP), graphene oxide (GO) and aniline (AN). Then, we studied the material properties under different ratios of the wave-transmitting agent and the wave-absorbing agent as well as the thicknesses of coatings.

CIP, with relatively broad bands, is one of the most important wave-absorbing materials and possesses three major absorption mechanisms: dielectric, resistive and magnetic loss [8]. It can absorb electromagnetic waves through polarization relaxation, hysteresis loss and natural resonance. GO has good electrical conductivity and excellent mechanical properties [9]. It has a sheet-like structure and a large specific surface area and can isolate the intrusion of corrosive media, such as water and oxygen. 

The surface has a large number of oxygen-rich functional groups [10], which can be more easily compounded with CIP and polyaniline. PANI is a conducting polymer with conjugated π-electrons. There are free radicals on the structure of polymer chains after doping, and the transition of dipoles makes the polymer conductive, so that a certain thickness of polyaniline coating can absorb electromagnetic waves. PANI is light, non-toxic, low-cost, and also possesses good electrochemical and reversible redox properties. The gel agglomeration can be solved to some extent by modifying PANI with dopants [11]. The unique anti-corrosion mechanism of PANI increases its application value [12,13], and it has become the most promising polymer used in anti-corrosion coatings.

**Figure 1:**
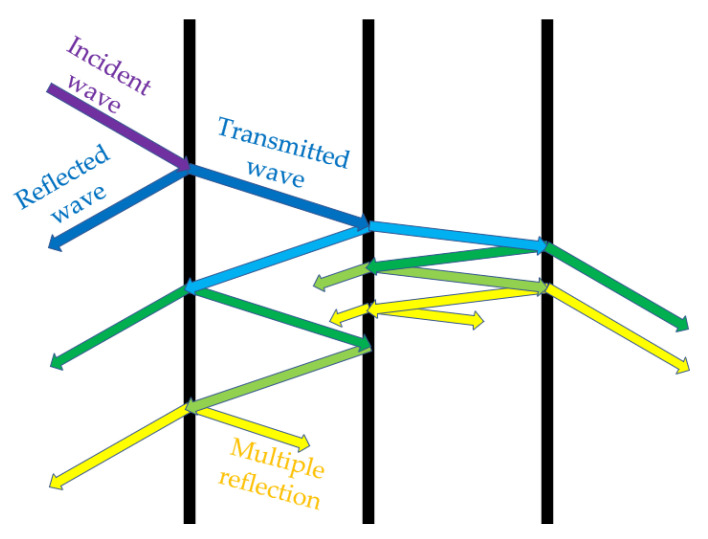
Schematic diagram of electromagnetic wave incidence.

Electromagnetic waves will inevitably encounter the interface of different media during the transmission process, and reflection and transmission phenomena occur at the interface. The principle of material transmission is shown in Figure 1 [14].

(1) T+R+A=1

(2) $$tan\delta =\frac{{A\lambda (\varepsilon }_{r}{-R}^{2}{\theta )}^{1/2}}{2\pi d\varepsilon }=\frac{W}{{RE}^{2}{f\varepsilon }_{r}}$$

where *R* is the power reflection coefficient, *A* is the attenuation coefficient, *T* is the transmission power coefficient, and the sum of the three is equal to 1 [15]. λ is the wavelength, tanδ is the loss tangent, θ is the angle of incidence, *d* is the thickness of the medium, *W* is the energy consumption for the conversion of electromagnetic energy into thermal energy, *f* is the frequency, *E* is the electric field strength, and $\varepsilon_{r}$ is the material relative dielectric constant. When the transmission medium is an ideal material, *R* does not change with frequency, at which the loss tangent of the electromagnetic wave is proportional to the attenuation coefficient *A*, the incidence angle θ, the wavelength λ, and the energy consumption *W* and inversely proportional to the media thickness *d*, the electric field strength *E*, the frequency *f*, and the relative dielectric constant $\varepsilon_{r}$.

(3) $$R_{L}=\frac{Z_{L}-Z_{0}}{Z_{L}{+Z}_{0}}$$

(4) $$Z_{L}=\sqrt{\frac{\mu }{\varepsilon }th(j\frac{2\pi }{\lambda }d\sqrt{\varepsilon \mu })}$$

**Figure 2:**
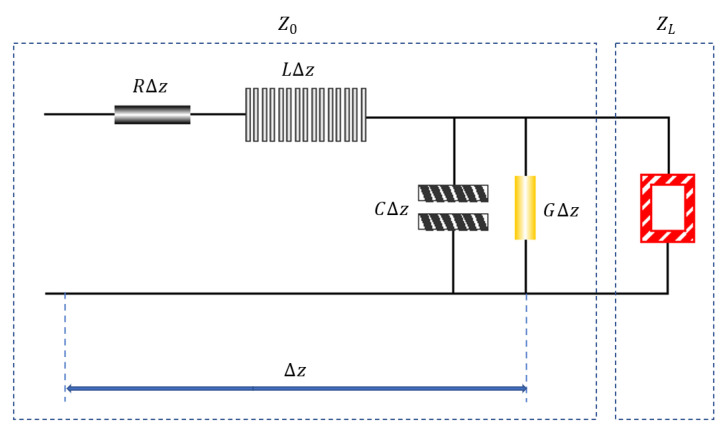
The principle of impedance matching.

Figure 2 shows the lumped parameter equivalent circuit of a line element ∆z on the transmission line, which consists of series resistor *R*, inductor *L*, parallel conductance G, and capacitor C. ε is the complex permittivity, μ is the complex permeability, $R_{L}$ is the normalized reflection coefficient, $Z_{L}$ is the load impedance, and $Z_{0}$ is the transfer line characteristic impedance. The electromagnetic wave transmission medium is equivalent to a section of load and transmission line [16,17], and the circuit input impedance changes with the position. 

When the load impedance $Z_{L}$ is equal to the characteristic impedance of the transfer line $Z_{0}$, the reflection coefficient $R_{L}=0$, at which point the electromagnetic wave belongs to the ideal traveling wave state, reaching the ideal zero reflection state. It can be seen that the absorption efficiency needs to be pursued on the premise that the surface impedance of the material is close to the spatial impedance. However, to satisfy the impedance match and to improve the absorption is often contradictory. 

Therefore, we need to adjust the ratio of the wave-transmitting agent and the wave-absorbing agent and the thickness of the coating. On one hand, sufficient resistivity is required to support impedance matching to reduce the reflection of electromagnetic waves on the incident surface. On the other hand, adequate dielectric properties and the magnetic conductivity are needed to support the absorbent performance to avoid the reflection of electromagnetic waves.

## 2. Materials and Methods

### 2.1. Materials

GO was purchased from Shenzhen Tulingjinhua Tech. Co., Ltd. (Shenzhen, China). CIP was purchased from Lijia Metal Co., Ltd. (Lijia, China). Concentrated hydrochloric acid (HCl) was obtained from Shenzhen Qihongyuan Technology Co., Ltd. (Shenzhen, China), sodium chloride (NaCl) from Yatai United Chemical Co., Ltd. (Wuxi, China), and ethanol (C_2_H_5_OH, 99.5%) from Linshi Chemical Reagent Co., Ltd. (Guangzhou, China). 

Aniline monomer (AN) and ammonium persulfate (APS) were purchased from Sinopharm Chemical Reagent Co., Ltd. (Shanghai, China). Epoxy resin (E-44) and curing agent (polyamide 650) were purchased from Liangli Electronic Commerce Co., Ltd. (Shanghai, China). The solvent was laboratory-prepared deionized water. The homogenizer was purchased from Changzhou Yineng Experimental Instrument Factory. The vector network analyzer was purchased from Keysight Technology (Qingdao, China) Co., Ltd.

### 2.2. Preparation of CIP-GO-PANI Nanocomposites

**Figure 3:**
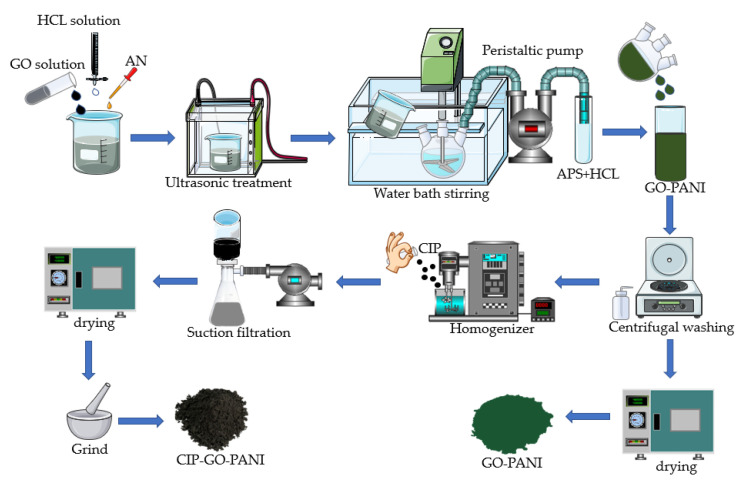
The schematic diagram of GO-PANI-CIP preparation.

Figure 3 shows the schematic diagram of GO-PANI-CIP preparation. The detailed preparation procedures are described as follows.

PANI-GO in-situ synthesis experiment: first, we added 3 g of aniline (0.20 mol) and 150 mL of dilute hydrochloric acid to 20 g of graphene oxide solution, stirred for 10 min, and ultrasonicated for 20 min. Next, we poured the GO-AN suspension into a 1 L three-necked flask in an ice bath at −5~0 °C and stirred it evenly. Then, 5 g of the APS solution (50 mL) was slowly added dropwise for 1 h [18]. Finally, the reaction was mixed by stirring for 24 h. After, we washed it repeatedly with deionized water and alcohol and put one part into a homogenizer. The other part was dried at 60 °C in a vacuum for 24 h.

CIP coating test: we added 1 L of GO-PANI suspension (150 g/L) to the homogenizer, add 300 g of CIP, stirred and coated for 12 h, and then moved it into a 60 °C oven for drying.

### 2.3. Characterization

The surface morphology and dispersion of the material were characterized by a scanning electron microscope (JSM-6360). The surface functional groups of the material were measured by Fourier transform infrared spectroscopy (Spectrum GX) with the scanning range of 4000–500 cm^−1^. X-ray Diffraction (XRD) with the wavelength of λ = 1.53 Å, a scan rate of 0.2 s, a voltage of 40 kV, and a scan range of 10–90° was used to test the particle size and structure characteristics of the material. 

The electromagnetic parameters and microwave absorbing properties of samples at 2–18 GHz were tested by a vector Network analyzer. The Tafel polarization curve and electrochemical impedance spectroscopy (EIS) of the material were tested by an electrochemical workstation (CHI660) to analyze the anti-corrosion performance.

## 3. Results and Discussion

### 3.1. SEM Analysis

**Figure 4:**
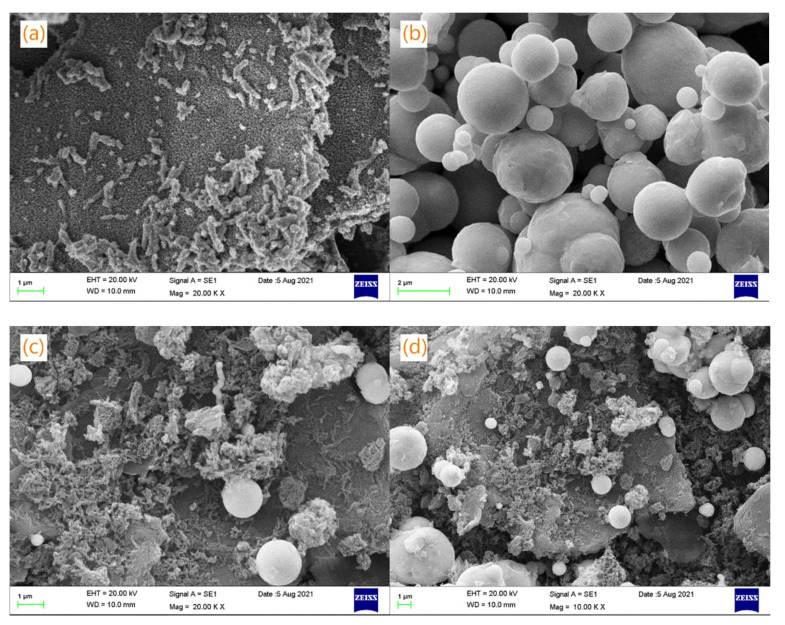
SEM images of (**a**) GO-PANI, (**b**) CIP, (**c**) and (**d**) CIP-GO-PANI (CIP:GO-PANI = 1:1).

Figure 4 shows the SEM images of the GO-PANI, CIP and CIP-GO-PANI composite materials. Metal ultrafine powder refers to powder with a particle size of 10 μm or even less than 1 micron. Due to the refinement of the particles, the number of atoms of the composed particles is greatly reduced, and the activity is greatly increased. As shown in Figure 4a, the GO-PANI composite material shows that PANI is in the shape of a wire rod and aggregates on the surface of the GO with a sheet structure, which is consistent with the literature [19]. 

The intercalation of GO with the agglomeration phenomenon is significantly reduced, and the dispersibility is clearly enhanced. The fiber network structure of PANI has changed. The results showed that the GO-PANI composite material was successfully synthesized. As shown in Figure 4b, the CIP is spherical. In Figure 4c,d, the spherical CIP successfully adhered to the sheet-shaped GO-PANI, indicating the successful polymerization of the CIP-GO-PANI composite. GO-PANI with good dispersibility is used as a coating agent and surfactant to inhibit the growth of CIP nanoparticles and avoid the agglomeration of CIP particles.

### 3.2. XRD Analysis

**Figure 5:**
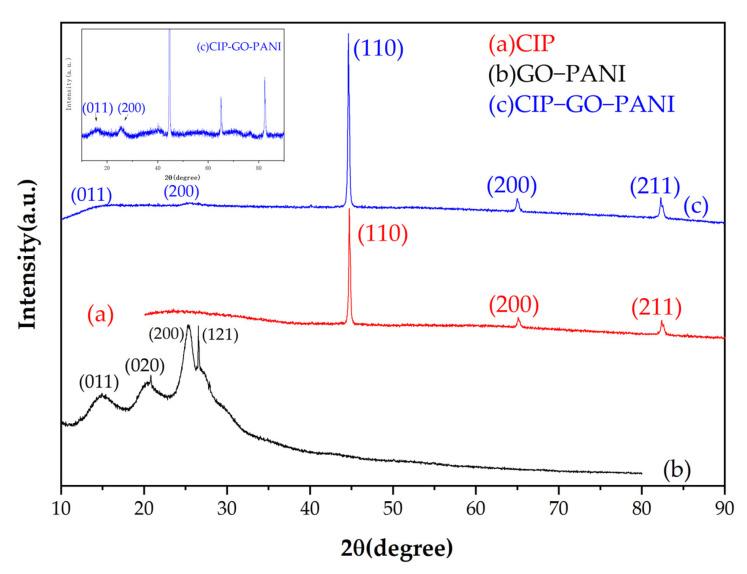
XRD images of (**a**) CIP, (**b**) GO-PANI and (**c**) CIP-GO-PANI.

As shown in Figure 5. In the XRD diffraction pattern of GO-PANI, it can be seen that there are clear characteristic peaks at 2θ = 15.2°, 20.3°, 25.3° and 26.5°, corresponding to GO-PANI’s (011), (020), (200) and (121) diffraction peaks of crystal planes on the diffraction plane [20,21]. This shows that the material structure is not destroyed during the synthesis process. The XRD diffraction pattern of the final synthesized product CIP-GO-PANI shows that the characteristic peaks of GO-PANI are still maintained at 15.2° and 25.3°; however, the intensity is weak, which may be because the surface is coated with CIP. The characteristic peaks of CIP are maintained at 44.6°, 65.0° and 82.3°, which shows that GO-PANI was successfully coated on the CIP surface, and the obtained composite material has the phase structure of the target product [22].

### 3.3. FT-IR Analysis

**Figure 6:**
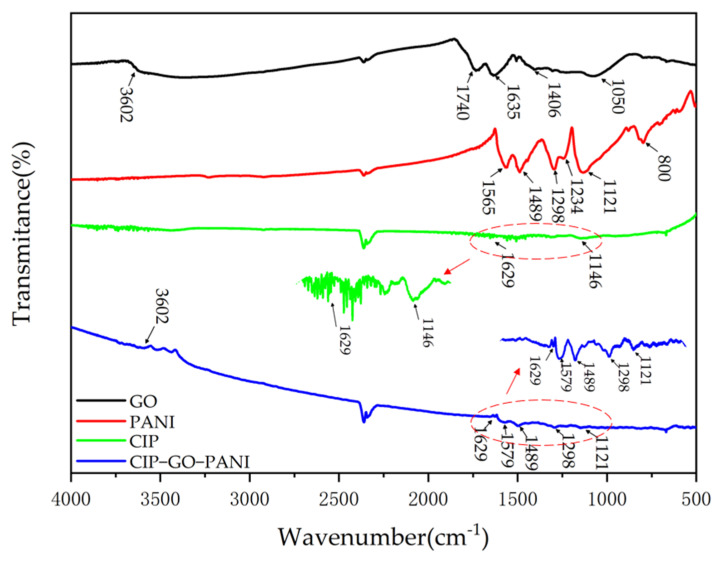
FT-IR images of GO, PANI, CIP and CIP-GO-PANI nanocomposites.

Figure 6 is the infrared spectrum of CIP-GO-PANI. For GO, the peak at 3602 cm^−1^ is the stretching vibration peak of the O-H group in the C-OH in GO, the peak at 1740 cm^−1^ is attributed to the C=O stretching vibration [23], and the peak at 1635 cm^−1^ is the C=C stretching vibration peak of the sp^2^ hybrid structure of GO. The peak at 1406 cm^−1^ is caused by the stretching vibration of C-OH, and the peak at 1050 cm^−1^ is the C-O-C stretching vibration peak of the carboxyl group [24]. 

For PANI, the peak at 1565 cm^−1^ is attributed to the C=C stretching vibration of quinone; the peak at 1489 cm^−1^ is caused by the C=C stretching vibration of the benzene ring; and the peak at 1298 cm^−1^ corresponds to the C-N-C (secondary amine group) stretching in the aromatic amine structure. The vibration peak at 1234 cm^−1^ is assigned to the C=N stretching vibration of aromatic amine, and the peaks at 1121 and 800 cm^−1^ are attributed to C-H in-plane and out-of-plane bending, respectively. 

The peaks at 1146 and 1629 cm^−1^ are the characteristic peaks of CIP. The peak at 2361 cm^−1^ is the characteristic peak of CO_2_. In the infrared spectrum of the CIP-GO-PANI composite material, it can be seen that the composite material still retains the characteristic peaks of 3602, 1629, 1579, 1489, 1289 and 1121 cm^−1^, indicating the successful synthesis of the composite materials.

### 3.4. Analysis of Electromagnetic Parameters

After washing CIP-GO-PANI with deionized water and alcohol twice to remove residual hydrochloric acid and putting it in a vacuum drying oven for drying, the composite was mixed evenly with epoxy resin and a curing agent in a beaker, heated and stirred, and then taken out while curing. After curing, the mixture was poured into a three-roll machine for grinding and then mixed with paraffin in a mass ratio of 3:7, making coaxial specimens with different thicknesses with an inner diameter of 3 mm and an outer diameter of 7 mm [25,26]. A vector network analyzer was used to test the electromagnetic parameters and wave absorbing performance of the material in the range of 2–18 GHz.

**Figure 7:**
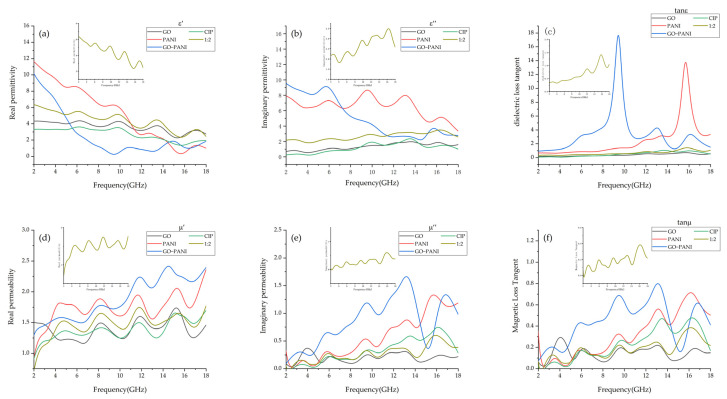
(**a**) Real permittivity, (**b**) imaginary permittivity, (**c**) dielectric loss tangent, (**d**) Real permeability, (**e**) imaginary permeability and (**f**) magnetic loss tangent of GO, PANI, GO-PANI, CIP and GO-PANI:CIP = X:Y (X and Y are the ratios in the Figure above).

From Figure 7a,c, we can roughly know that the higher the initial polarization degree, the greater polarization relaxation loss when the frequency increases. Comparing different ratios of composite materials, it can be seen that, with the increase of the CIP ratio, the real part of the high-frequency dielectric gradually increases. The dielectric real part of GO-PANI is larger at low frequency, which indicates that GO-PANI has a greater degree of polarization and a higher conductivity at low frequencies. Therefore, in the follow-up test at low frequencies, GO-PANI exhibits reflection loss (RL), while CIP has greater polarization and higher conductivity at high frequencies [27], which helps CIP act as a dielectric wave absorber at high frequencies. 

From Figure S1a and Figure 7a, the real permittivity of composite materials with ratios of 8:1 and 4:1 at low and medium frequencies is higher than that of GO-PANI and CIP, while composite materials with a ratio of 1:16 also have the highest dielectric real part at high frequencies. It can be seen that the dielectric loss of the coated composite material at each frequency is greater than that of the pure component, which helps to improve the wave-absorbing performance of the composite material. 

As shown in Figure 7b, it can be seen that the imaginary permittivity increases after the combination of GO and PANI, and the resistive loss becomes larger. From the perspective of the proportional relationship between GO-PANI and CIP, the higher the GO-PANI and PANI content, the greater the resistive loss, indicating that they occupy a major position in the resistive loss. We found that the imaginary permittivity of the composite material with strong conductivity decreases to some extent at medium and high frequencies. It may be due to a too large conductance. The skin effect of the conductive mesh occurs at high frequencies and without being conducted to the inside of the material, and thus the loss is reduced.

The dielectric loss tangent *tanδ* represents the overall dielectric loss. The larger the loss angle *δ*, the larger *tanδ*, and the electromagnetic energy is converted into heat.

(5) $${W=R\cdot E}^{2}{f\varepsilon }_{r}tan\delta$$

where *W* is the energy consumption, *R* is the coefficient, *E* is the electric field strength, *f* is the frequency, $\varepsilon_{r}$ is the relative permittivity of the material, and *δ* is the loss angle. As shown in Figure 7c, *tanδ* generally shows an upward trend as the frequency increases. After GO and PANI are combined, *tanδ* increases in the middle and low frequencies zone. Thus, the absorption performance of the middle and low frequency is improved, and the performance of the high frequency should be analyzed from the magnetic loss.

Combined with the RL test, it can be seen that, for the prepared CIP-GO-PANI composite material, the magnetic loss of CIP dominates at high frequencies mainly from hysteresis and resonance loss [28].

As shown in Figure 7d, the real permeability generally does not change much, with a slow increase. It can be seen that as the frequency increases, the electromagnetic induction in the material gradually increases, and the induced current generates an opposite magnetic field that prevents the external magnetic flux from changing. The opposite magnetic field causes the actual magnetic field to be weakened, that is, the hysteresis loss causes the attenuation of electromagnetic waves [29]. 

It can be seen from the ratio that the hysteresis loss increases after GO and PANI are combined, and the higher content of GO-PANI, the greater magnetic hysteresis loss. It has peaks of 1.77, 2.24 and 2.41 at 8.15, 11.82 and 14.53 GHz, respectively. As shown in Figure 7e, the imaginary permeability rises with increasing frequency. The magnetic loss tangent indicates the overall magnetic loss of the material. As shown in Figure 7f and Figure S1f, as the frequency increases, tanμ shows an overall upward trend, and the peak positions are the same as the peak positions of tanδ. It can be seen that the coated material has the largest magnetic loss at a high frequency, which is similar to the situation in the literature [30].

### 3.5. Reflex Loss Curve Analysis

**Figure 8:**
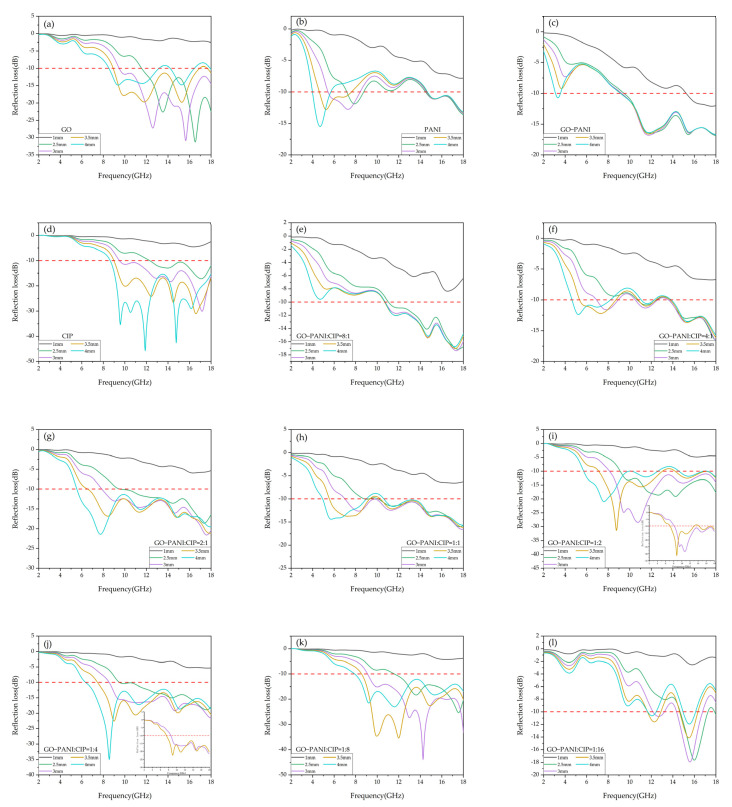
RL curves of (**a**) GO, (**b**) PANI, (**c**) GO-PANI, (**d**) CIP, (**e**) GO-PANI:CIP = 8:1, (**f**) PANI:CIP = 4:1, (**g**) GO-PANI:CIP = 2:1, (**h**) GO-PANI:CIP = 1:1, (**i**) GO-PANI:CIP = 1:2, (**j**) GO-PANI:CIP = 1:4, (**k**) PANI:CIP = 1:8 and (**l**) GO-PANI:CIP = 1:16.

As shown in Figure 8a and Figure S2a, the wave absorbing performance is improved when the thickness of the GO coating reaches 2 mm. The absorption performance reaches the highest when the thickness is 2.5–3 mm. The maximum bandwidth is 9.46–18.00 GHz at 3 mm, with peaks of −27.20 and −30.83 dB at 12.57 and 15.64 GHz. When the thickness is higher than 3.5 mm, the absorbing effect gradually decreases. It can be seen that GO has certain absorbing properties [31]. Since GO is expensive, it cannot be used as the main material.

However, a small amount of flake GO has a good anti-corrosion effect. The main purpose of choosing GO in this topic is to achieve the anti-corrosion effect as well. As shown in Figure 8b,c and Figure S2b,c, both have wave-absorbing performance at high and low frequencies for PANI and GO-PANI; however, the performance at low frequencies is only for the thickness of more than 4 mm, and thus it is of little practical significance. As shown in Figure 8d, for CIP, the ideal thickness is 3 or 3.5 mm. 

CIP not only has a wide frequency band and is distributed in the middle and high frequency bands but also has an excellent wave absorbing effect. It is a good medium and high frequency wave absorber [32]. When the thickness is 3 mm, the absorption is higher than −10 dB for frequency bands between 9.51 and 18 GHz, the peak is −30.06 dB at 17.18 GHz, and the absorption rate reaches 99%. With the sample higher than 3 mm, the bandwidth distribution and peak value are approximately the same as those at 3 mm.

If the thickness increases further, the performance will decrease. As shown in Figure 8e–h, the properties of the composites in this ratio are basically similar to those of pure PANI. At low frequencies, approximately from 3.12 to 9.41 GHz, absorption is still effective, but the thickness corresponding to impedance matching is too large for practical significance. In addition, there is a certain wave absorbing performance at high frequencies, and the peak position is almost the same. It can be seen that, although GO-PANI has wave-absorbing properties at low frequencies, a higher thickness is required for impedance matching. If the material impedance can be adjusted effectively, the GO-PANI will be a high-efficiency absorbing agent at low frequencies.

As shown in Figure 8i,j, the composite materials with the current ratio demonstrated excellent wave-absorbing performance at high frequencies. The absorbing performance first increases and then decreases with the increasing thickness. The matching frequency gradually moves towards the low frequency direction [33]. When the ratio reaches 1:8 in Figure S2k, the material lost its low-frequency absorbing performance; however, after 12.0 GHz, it shows strong absorption and has a larger bandwidth of 2.5–5 mm. 

It also has the best absorbing performance at thicknesses between 3 and 3.5 mm. For example, at 3 mm, the frequency band is distributed from 9.10 to 18.00 GHz, and the maximum RL is −43.33 dB at 14.27 GHz. When the ratio reaches 1:16 in Figure 8l, the material’s absorbing performance is greatly reduced, and the frequency bandwidth and the peak are reduced.

For the RL of composite materials at a certain thickness, a certain amount of GO-PANI can improve the absorbing performance of the material at low frequencies. When the proportion of GO-PANI is increased, the bandwidth is increased as well and the peak moves to the low frequency direction correspondingly. The material demonstrated better performance when the thickness was between 3 and 3.5 mm [34], and the best ratio of GO-PANI to CIP is 1:2. 

The frequency band is distributed from 7.12 to 18.00 GHz, the peak value is −31.29 dB, and the maximum RL is −31.29 dB at 8.77 GHz. The situation for the thickness of 4 and 4.5 mm is almost the same as that of 3 mm, but a too-thick coating is expensive. When the thickness reaches 5 mm, not only is the thickness is too large but also the absorbing performance at high frequency is greatly reduced, and the RL at multiple points is only 90%.

### 3.6. Electrochemical Measurement

For preparing samples, the following materials, GO, PANI, GO-PANI, CIP, GO-PANI-CIP (GO-PANI:CIP = 1:2) and GO-PANI-CIP (GO-PANI:CIP = 2:1) were stirred and dispersed in the curing agent. After the materials were dispersed uniformly, epoxy resin was added and stirred continuously. The as-prepared suspension was spread on the working electrodes that has been polished using 500-mesh, 1000-mesh and 2000-mesh sandpapers and cleaned with alcohol in advance. The working electrode had a surface area of 1 × 1 cm made of Q215 stainless steel only.

All coverage of the same electrode was measured. The reference electrode was an AgCl electrode. The working electrode was put into a 3.5 wt% NaCl solution for 3600 s [35] to test the open circuit potential. If the potential fluctuation did not exceed 0.5 mV in the last 10 min, we used an electrochemical workstation to measure the impedance spectroscopy and potentiodynamic polarization curve [36,37]. The start frequency was 10^5^ Hz, the end frequency was 10^−2^ Hz, the amplitude was 0.005 V, and each measurement frequency was 10 points, for a total of 70 points.

#### 3.6.1. Electrochemical Impedance Spectroscopy (EIS)

**Figure 9:**
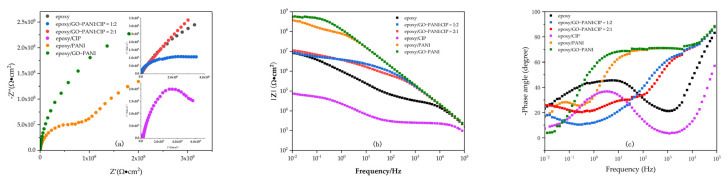
Nyquist plots (**a**) and Bode plots (**b**,**c**) for epoxy, CIP, PANI, GO-PANI and CIP/GO-PANI composites with different ratios.

In this section, EIS was used to study the anti-corrosion behavior for the six coatings. The Nyquist spectrum of different material coatings is shown in Figure 9a. The horizontal axis is the real impedance part, and the vertical axis is the imaginary part. Each point of the Nyquist spectrum corresponds to a different measurement frequency. The radius of the arc indicates the anti-corrosion performance of the material [38]. The larger the diameter is, the larger the impedance modulus. The smaller the corrosion current density is, the better the corrosion resistance. 

In the figure, the order of diameter size is epoxy/GO-PANI > epoxy/PANI > epoxy/GO-PANI:CIP = 2:1 > epoxy/GO-PANI:CIP = 1:2 > epoxy > epoxy/CIP. The comparison of the curves of epoxy/PANI and epoxy shows that the addition of PANI to epoxy resin can increase its anti-corrosion performance. According to Figure 9b, it can be seen that its modulus reaches nearly 3.88 × 10^8^ Ω·cm^2^, which benefits from the good electrochemical and reversible redox characteristics of the PANI. 

The coating has physical and chemical dual anti-corrosion effects. Comparing the curves of the epoxy/PANI and epoxy/GO-PANI, it can be seen that the anti-corrosion performance is improved after adding a certain amount of GO, reaching 5.57 × 10^8^ Ω·cm^2^, due to the flake structure of GO and the large specific surface area. 

This can isolate the intrusion of corrosive media, such as water and oxygen, to improve its anti-corrosion performance. It can be seen from the epoxy/CIP and epoxy/GO-PANI/CIP curves that, although epoxy/CIP has poor anti-corrosion performance, the impedance of the CIP coated with GO-PANI increased from less than 10^5^ Ω·cm^2^ to more than 10^7^ Ω·cm^2^, and the anti-corrosion performance is greatly enhanced. 

**Figure 10:**
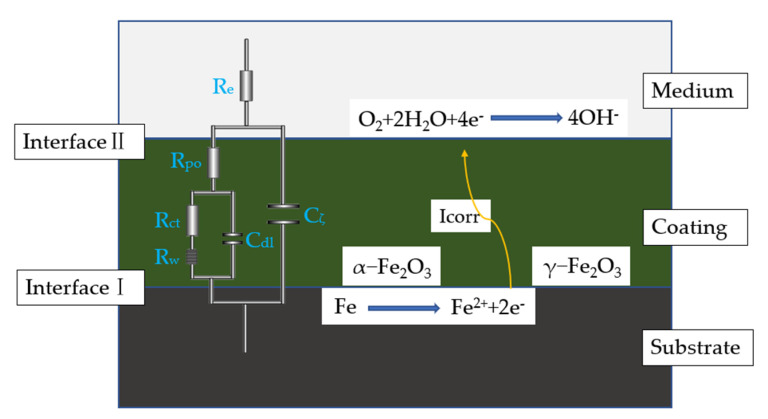
Separation of the anode and cathode reactions in the metal corrosion process with coatings (right)/equivalent circuit (left).

This is because the GO-PANI coating densely covers the working electrode, thereby, preventing the intrusion of ${\mathrm{OH}}^{-}$ and other anions. When FE^2+^ reaches a certain concentration, the reverse reaction is intensified, and the corrosion reaction is inhibited. The formed iron oxide passivation film prevents the intrusion of corrosive media and further improves the anti-corrosion performance. The anti-corrosion mechanism is shown in Figure 10.

The Bode diagram is shown in Figure 9b. The medium and high frequency region of the impedance modulus curve responds to the corrosion reaction between corroded materials and metals. The low frequency area responds to the protection of the coating, and the |Z| determines the corrosion resistance of the coating. The Figure shows that the order of the curve impedance modulus is: epoxy/GO-PANI > epoxy/PANI > epoxy/GO-PANI:CIP = 2:1 > epoxy/GO-PANI:CIP = 1:2 > epoxy > epoxy/CIP. 

The modulus of the GO-PANI/CIP coating is increased by two orders of magnitude compared with that of the CIP coating. The improvement of the anti-corrosion performance of the Bode diagram is essentially consistent with the conclusion of the impedance spectrum as shown in Figure 9c. At high frequencies, GO-PANI has a larger phase shift indicating better capacitive behavior. After CIP is coated with GO-PANI, the phase shift is also greatly increased.

As shown in Figure 10, ZSimpWin was used to fit the EIS of the GO/PANI:CIP = 1:2 sample with the best comprehensive performance, and the corresponding equivalent circuit is supplemented in the original Figure 10. R_e_ is the solution resistance, R_po_ is the pore resistance, R_ct_ is the charge transfer resistance, C_ζ_ is the coating capacitance, and C_dl_ is the electric double layer capacitance. In addition, R_w_ is the diffusion resistance of the electrode, which characterizes the diffusion behavior of the coating. The fitting curve is shown in Figure S3.

#### 3.6.2. Potentiodynamic Polarization Studies

**Figure 11:**
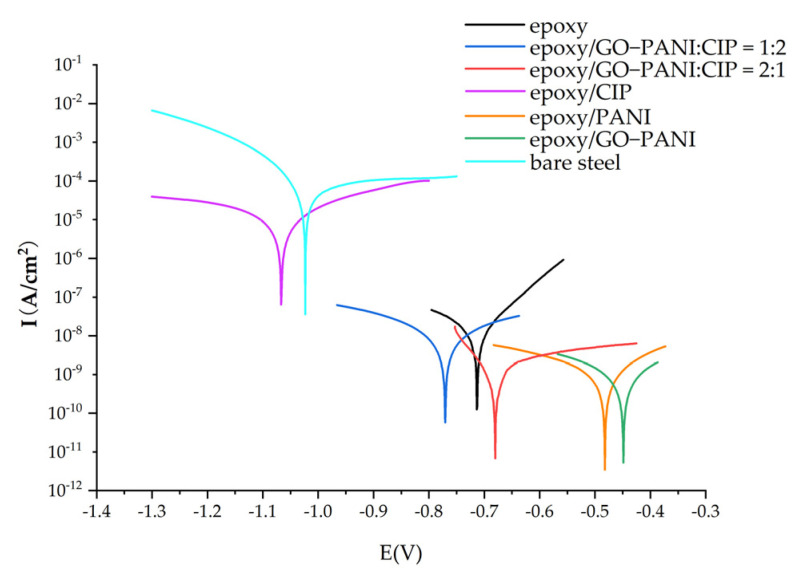
Potentiodynamic polarization curves for the different coatings.

Figure 11 shows the potentiodynamic polarization curve of each electrode in a 3.5 wt% NaCl solution. The horizontal axis is the potential vs. reference electrode, and the vertical axis is the current. The intersection of the cathode and anode polarization curves is the corrosion potential (Ecorr) and corrosion current (Icorr) of the material. The corrosion potential, corrosion current and corrosion rate (CR) of each material are shown in Table 1.

The corrosion resistance of the composite material is analyzed from the corrosion potential and the corrosion current. The corrosion potential Ecorr mainly represents the tendency of corrosion reaction. The higher Ecorr of the sample coating, the higher corrosion threshold [39] and the stronger adaptability to the environment. It can be seen in Figure 11 that the corrosion potential of the epoxy coating after adding PANI and GO-PANI materials has a positive displacement, reaching −0.4 to −0.5 V. Bare steel and epoxy/CIP coating have a lower corrosion potential. After being compounded with GO-PANI, the corrosion potential is increased to −0.77 and −0.68 V, and the effect of improvement is related to the content of GO/PANI [40].

The relationship between corrosion current and corrosion rate is

(6) $$CR=\frac{{KMI}_{corr}}{\rho V}$$

where *K* is 3268.5 (mol/A), *M* is the molecular weight of iron 56 (g/mol), *ρ* is the density of iron (7.85 g/cm^3^), *V* is the valence of iron [41], and the corrosion rate is proportional to the corrosion current. The PANI coating and GO-PANI coating have smaller corrosion currents: 1.99 × 10^−9^ A/cm^2^ and 1.67 × 10^−9^ A/cm^2^, respectively. The corrosion current of the CIP coating is 5.13 × 10^−5^ A/cm^2^, but the corrosion current of the coating after CIP coated with GO-PANI is reduced to about 10^−9^ A/cm^2^. The corrosion current is reduced by four orders of magnitude, and the anti-corrosion performance is greatly enhanced. The higher the proportion of GO-PANI is, the better the effect. In this case, the corresponding corrosion rate is only 2.67 × 10^−5^ mm/year.

It can be seen that coating a certain amount of GO-PANI on the CIP surface could increase the corrosion potential, reduce the corrosion current, enhance the anti-corrosion performance, and improve the medium and low-frequency wave absorbing performance.

## 4. Conclusions

An in-situ chemical polymerization and a physical coating were used to prepare a composite material CIP-GO-PANI with the dual properties of electromagnetic wave absorption and corrosion resistance. The results show that, on the basis of the strong wave-absorbing performance of CIP, the coated CIP-GO-PANI composite material not only has a greater absorbing wave bandwidth compared with CIP but also has a higher peak at low frequencies than CIP. 

The best result is achieved when the thickness of coating is 3–3.5 mm and the ratio of GO-PANI:CIP is 1:2. After coating with GO-PANI, the impedance increases from less than 10^5^ to more than 10^7^, the corrosion potential is increased to −0.7 V, the corrosion current is reduced to 10^−9^, and the corrosion rate is reduced by five orders of magnitude. In general, the CIP-GO-PANI composite material has massive advantages, such as simple preparation, stable performance, effective corrosion protection and high electromagnetic wave absorption.

## Figures and Tables

**Table 1:** The Tafel plot data for the different coatings.

Sample	Ecorr (V)	Icorr (A/cm^2^)	Corrosion Rate (mm/Year)
Bare steel	−1.023	1.58 × 10^−4^	1.85 × 10^−0^
Epoxy	−0.712	1.39 × 10^−8^	1.63 × 10^−4^
Epoxy/PANI	−0.483	1.99 × 10^−9^	2.33 × 10^−5^
Epoxy/GOPANI	−0.450	1.67 × 10^−9^	1.96 × 10^−5^
Epoxy/CIP	−1.067	5.13 × 10^−5^	0.60 × 10^−0^
Epoxy/GO-PANI:CIP = 1:2	−0.772	6.71 × 10^−8^	7.87 × 10^−4^
Epoxy/GO-PANI:CIP = 2:1	−0.680	2.28 × 10^−9^	2.67 × 10^−5^

## Data Availability

Data sharing is not applicable to this article.

## Supplementary Materials

The following supporting information can be downloaded at: https://www.mdpi.com/article/10.3390/ma15020484/s1, Figure S1: (a) Real permittivity, (b) Imaginary permittivity, (c) Dielectric loss tangent, (d) Real permeability, (e) Imaginary permeability and (f) Magnetic loss tangent of GO, PANI, GO-PANI, CIP and GO-PANI:CIP = X:Y (X and Y are the ratios in the Figure above); Figure S2: RL curves of (a) GO, (b) PANI, (c) GO-PANI, (d) CIP, (e) GO-PANI:CIP = 8:1, (f) PANI:CIP = 4:1, (g) GO-PANI:CIP = 2:1, (h) GO-PANI:CIP = 1:1, (i) GO-PANI:CIP = 1:2, (j) GO-PANI:CIP = 1:4, (k) PANI:CIP = 1:8 and (l) GO-PANI:CIP = 1:16; Figure S3: Analysis of the EIS data using an equivalent circuit model; Table S1: The Tafel plot data for the different coatings.
