# Supplementary material for: Preparation and Characterization of Graphene Oxide/Polyaniline/Carbonyl Iron Nanocomposites
Source: Materials (Basel). 2022 Jan 9;15(2):484. doi: 10.3390/ma15020484 (PMC8781392; doi:10.3390/ma15020484)
Supplement: Supplementary file 1 [file materials-15-00484-s001.zip › materials-1489794-supplementary.pdf]

materials

MDPI

Article

Preparation and Characterization of Graphene Oxide/Polyaniline/Carbonyl Iron Nanocomposites

Yun-Yun Huang * and Jian Wu

College of Chemical Engineering, Fuzhou University, Fuzhou 350108, China; N190420034@fzu.edu.cn
* Correspondence: huangyunyun@fzu.edu.cn; Tel.: +86-13850119011

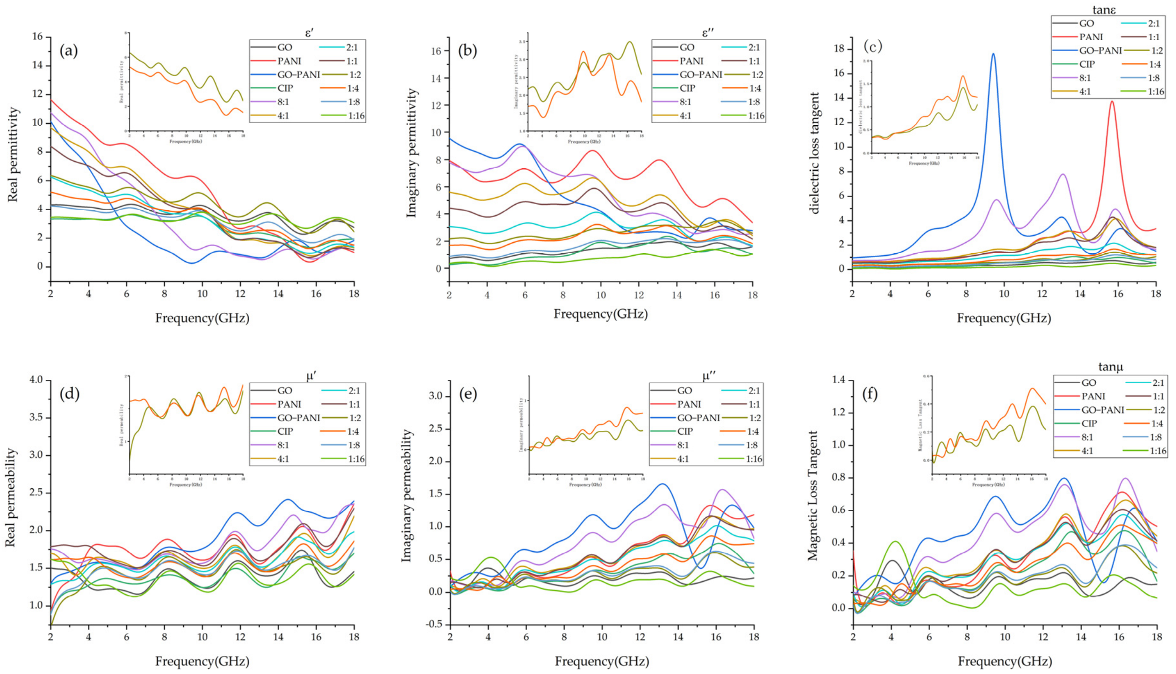

Figure S1. (a) Real permittivity, (b) Imaginary permittivity, (c) Dielectric loss tangent, (d) Real permeability, (e) Imaginary permeability and (f) Magnetic loss tangent of GO, PANI, GO-PANI, CIP and GO-PANI:CIP=X:Y(X and Y are the ratios in the Figure above).

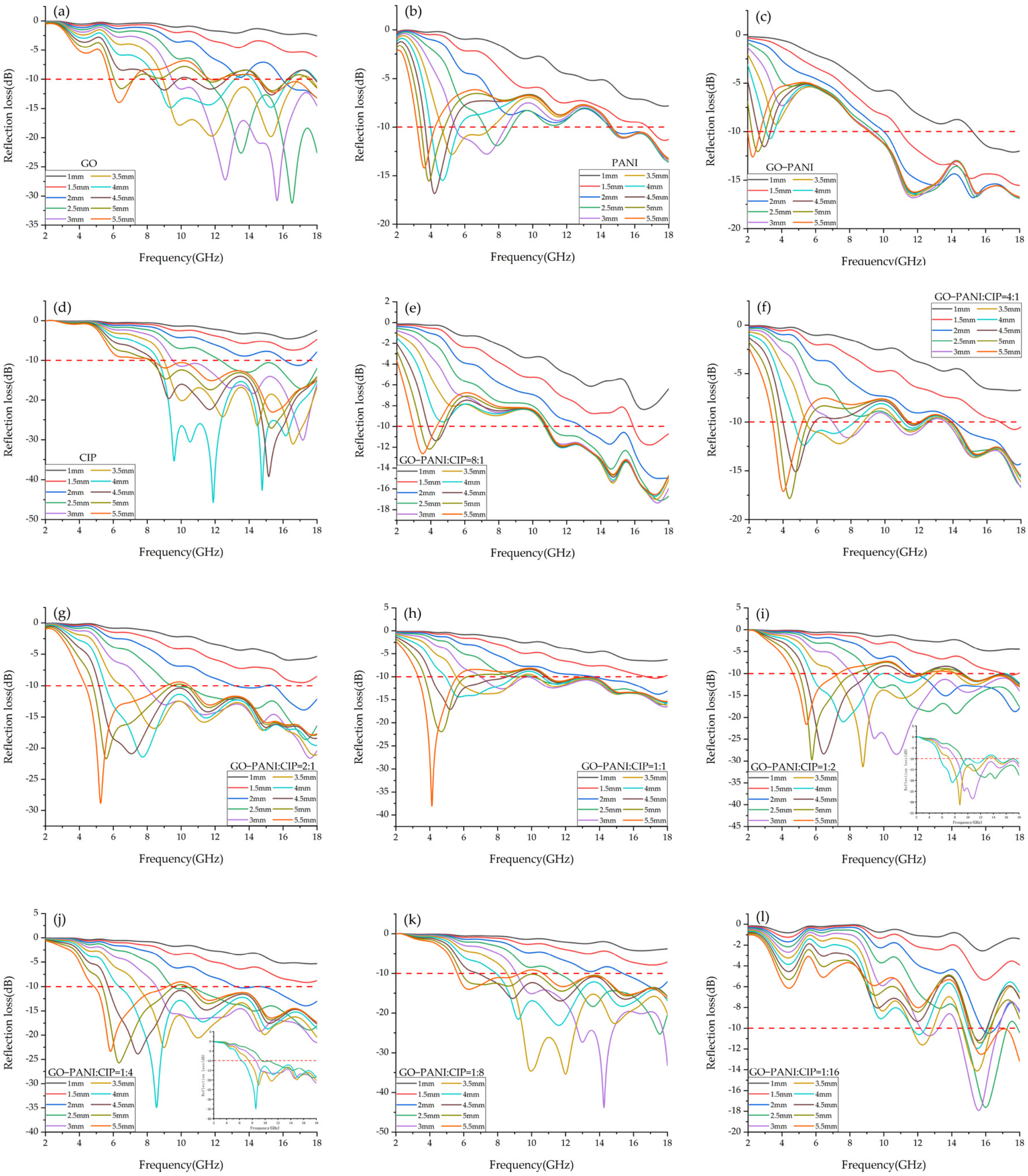

Figure S2. RL curves of (a) GO, (b) PANI, (c) GO-PANI, (d) CIP, (e) GO-PANI:CIP=8:1, (f) PANI:CIP=4:1, (g) GO-PANI:CIP=2:1, (h) GO-PANI:CIP=1:1, (i) GO-PANI:CIP=1:2, (j) GO-PANI:CIP=1:4, (k) PANI:CIP=1:8 and (l) GO-PANI:CIP=1:16.

Table S1. The Tafel plot data for the different coatings.

Sample	Ecorr(V)	Icorr(A/cm^2)	Corrosion rate (mm/year)
Bare steel	−0.988	1.58×10^{-4}	1.85×10^{-0}
	−0.962	1.59×10^{-4}	1.86×10^{-0}
Epoxy	−0.712	1.39×10^{-8}	1.63×10^{-4}
	−0.672	1.37×10^{-8}	1.60×10^{-4}
	−0.751	1.31×10^{-8}	1.53×10^{-4}
Epoxy/PANI	−0.483	1.99×10^{-9}	2.33×10^{-5}
	−0.476	2.11×10^{-9}	2.47×10^{-5}
	−0.468	1.85×10^{-9}	2.17×10^{-5}
Epoxy/GOPANI	−0.450	1.67×10^{-9}	1.96×10^{-5}
	−0.455	1.57×10^{-9}	1.84×10^{-5}
	−0.396	1.69×10^{-9}	1.98×10^{-5}
Epoxy/CIP	−1.067	5.13×10^{-5}	0.60×10^{-0}
	−0.952	4.68×10^{-5}	0.54×10^{-0}
	−1.233	5.08×10^{-5}	0.59×10^{-0}
Epoxy/GO−PANI:CIP = 1:2	−0.772	6.71×10^{-8}	7.87×10^{-4}
	−0.806	7.06×10^{-8}	8.27×10^{-4}
	−0.824	6.55×10^{-8}	7.67×10^{-4}
Epoxy/GO−PANI:CIP = 2:1	−0.680	2.28×10^{-9}	2.67×10^{-5}
	−0.688	2.19×10^{-9}	2.56×10^{-5}
	−0.732	2.42×10^{-9}	2.83×10^{-5}

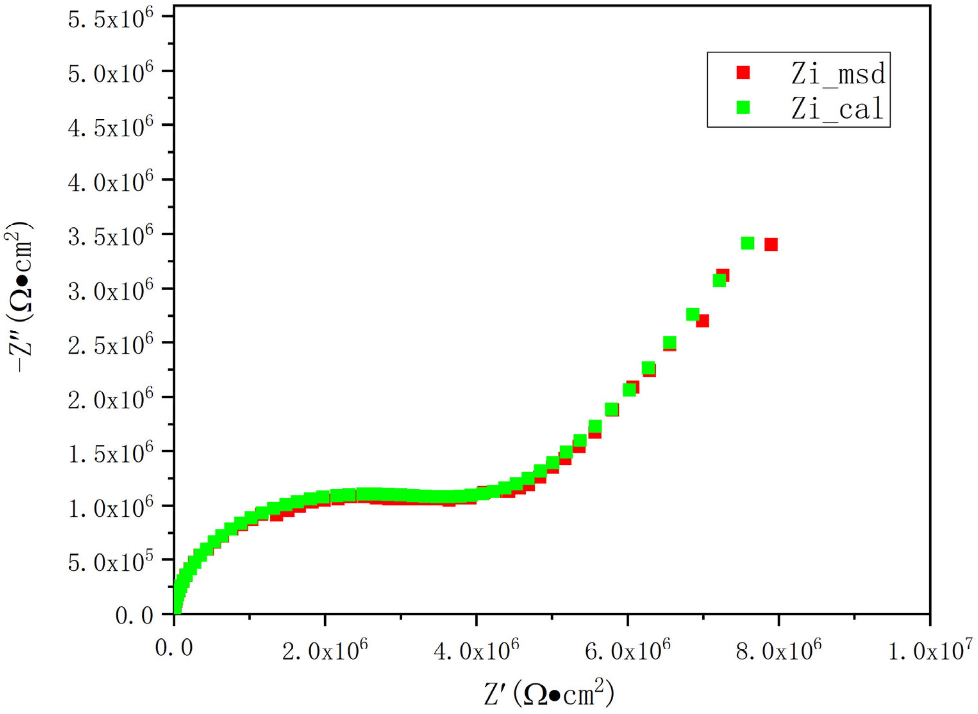

Figure S3. analysis of the EIS data using an equivalent circuit model.
